# Gas and Liquid Isotherms: The Need for a Common Foundation

**DOI:** 10.1021/acs.langmuir.4c04324

**Published:** 2025-01-21

**Authors:** Seishi Shimizu, Nobuyuki Matubayasi

**Affiliations:** † York Structural Biology Laboratory, Department of Chemistry, University of York, Heslington, York YO10 5DD, United Kingdom; ‡ Division of Chemical Engineering, Graduate School of Engineering Science, Osaka University, Toyonaka, Osaka 560-8531, Japan

## Abstract

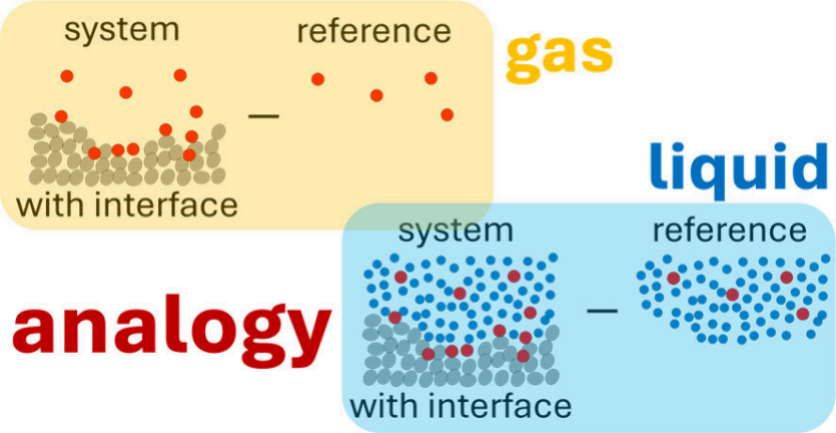

Sorption isotherms for gases and liquids have long been formulated separately. There is a fundamental problem with this approach: the popular isotherm models (such as Langmuir, BET, and GAB) for gases cannot be applied straightforwardly to sorption from solution. This contrasts with the theory of liquid solutions, where solute–solute interaction, mediated by the solvent, is captured as the potential of mean force, providing powerful interpretive tools (e.g., virial expansion) founded on the gas-liquid analogy. This analogy will be extended to sorption by adopting sorbate numbers and their fluctuations as the common foundation. This enables the gas and liquid isotherm equations to have an analogous mathematical form with a universal language for interfaces and liquid solutions.

## Introduction

“Adsorption from liquid solution is almost a new world in comparison with adsorption from the gas phase: the fundamental principles and methodology are different in almost all respects.”^1^ This view is based on the following well-established restrictions: (i) even the simplest isotherm models for gas adsorption (e.g., the Langmuir and Freundlich models), strictly speaking, cannot be applied directly to liquid adsorption;^1−3^ and (ii) the “individual” isotherm for sorbate, reported routinely for gas sorption,^4^ cannot be determined without employing “overly simplified models” like the “surface phase”.^2^

Such a gulf between gas and liquid adsorption contrasts with the study of liquid solutions where the gas–liquid analogy has long become a standard tool for analysis, starting from the textbook analogy between the ideal gas and van’t Hoff equation, culminating in the virial expansion for gases and liquid solutions.^5−11^ The basis of this analogy is the molecular distribution function as the measure of solute–solute interactions mediated by the solvent,^5−11^ which serves as a universal language for molecular thermodynamics, scattering, and computer simulation.^12,13^

Thus, the universality attained by the theory of liquids contrasts with the need for separate adsorption theories for gases and liquids; while the most common gas isotherm models assume site-specific, layer-by-layer binding on a uniform surface, liquid isotherm models involve hypothetical thickness, composition, or sorbate partitioning for introducing the “surface phase”.^1−3^ Because the gas and liquid isotherm models are overly idealized (as has long been recognized^14^), the mechanistic insights available from analyzing experimental isotherms have been strictly limited.

This Perspective aims to overcome these limitations and to provide a unified sorption theory encompassing vapor/solid and solution/solid interfaces. As the first step, the restrictions that necessitated gas and liquid sorption to be analyzed differently (see the opening paragraph) have been lifted recently by the statistical thermodynamic fluctuation theory.^15^ The “individual” isotherm can now be determined without introducing any models, by supplementing the surface excess isotherm (i.e., a competition between sorbate and solvent isotherms) with volumetric measurements.^16^ The isotherms for sorption from solution have been derived directly from the fluctuation theory with a clear physical meaning provided for their parameters.^15,17^ Moreover, statistical thermodynamic isotherms, derived for gases and liquids, have an analogous mathematical form.^18,19^

Thus, it is timely to re-examine whether “the fundamental principles and methodology are different in almost all respects”^1^ between the sorption of gases and liquid solutions. The objectives of this Perspective are

I. to establish the fundamental equations for gas and liquid sorption analogously;
II. to show that the isotherm equations for gas and liquid, derived directly from I, are analogous in form and interpretation; and
III. to demonstrate that the isotherm equations for gas and liquid can be simplified to yield the “surface phase”-based interpretation in a parallel manner.

Through these steps, sorption isotherms will attain the same degree of gas–liquid analogy as solution theory, which will facilitate isotherm analysis significantly.

## Sorption of Gases and Solutions Must Have Analogous Foundations

### Surface Excesses

**Figure 1:**
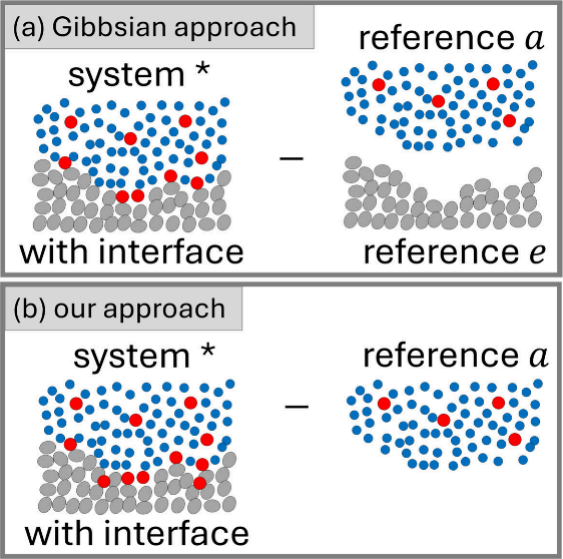
(a) The traditional Gibbsian setup for sorption from solution, involving sorbent (*e*), solvent (1), and sorbate (2) as the difference in molecular distribution between the system (denoted by *, which contains the interface) and the reference systems (without the interface) on the sorb*e*nt (denoted as *e*) and sorb*a*te (denoted as *a*) sides. (b) Our novel approach to defining the interfacial effect on the molecular distribution as the difference between the system (*) and the reference system on the sorbate side (*a*), which is conducive to a unified treatment of gas/solid and liquid/solid isotherms, incorporating adsorption and absorption, and reflecting the standard experimental practice for isotherm measurements (see main text for discussion). For a vapor/solid system, the bulk reference system *a* is a vapor consisting only of species 2. Note that the sorbate, sorbent, and solvent molecules can be of any size and shape; the spherical representation has been adopted here merely for simplicity.

Our first objective is to formulate the sorption of gases and solutions in an analogous manner (Objective I). To this end, it is imperative to introduce a consistent set of notations for gas/solid and solution/solid systems. Let *e*, 1, and 2 be the indexes for sorb*e*nt, solvent, and sorbate molecules, respectively. The traditional Gibbsian setup (Figure 1) involves a system (denoted by *, which contains the interface), as well as the reference systems without the interface on the solid sorb*e*nt side (denoted as *e*) and the sorb*a*te side (denoted as *a*); the bulk reference system *a* is a vapor consisting only of species 2 for a vapor/solid system and a solution comprised of species 1 and 2 for a solution/solid system (Figure 1).

**Figure 2:**
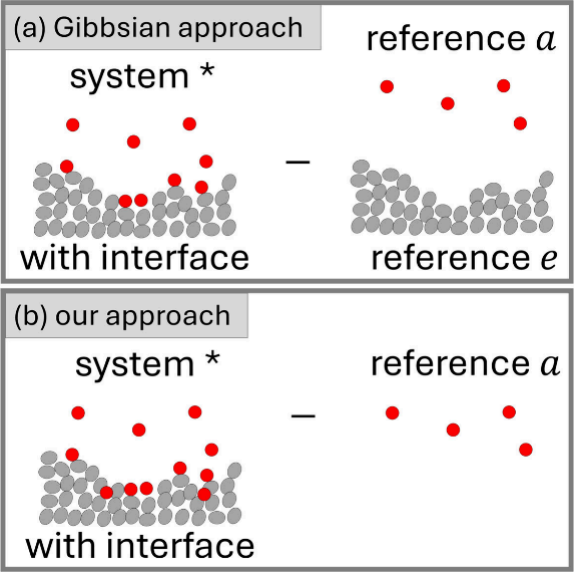
(a) The traditional Gibbsian setup for gas sorption, involving sorbent (*e*) and sorbate (2) as the difference in molecular distribution between the system (denoted by *, which contains the interface) and the reference systems (without the interface) on the sorb*e*nt (denoted as *e*) and sorb*a*te (denoted as *a*) sides. (b) Our novel approach to defining the interfacial effect on the molecular distribution as the difference between the system (*) and the reference system on the sorbate side (*a*). See the main text for how our approach enables a seamless connection between gas/solid and liquid/solid sorption isotherm theories.

For the clearest manifestation of the analogy between vapor/solid (Figure 2) and solution/solid (Figure 1) isotherm theories, we propose to define vapor/solid surface excess, Γ_2_, via
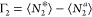
1aas the difference in sorbate number between the system ⟨*N*_2_^*^⟩ and the reference system ⟨*N*_2_^*a*^⟩ (Supporting Information: Section A). There is a subtle yet important difference between this definition (eq 1a) and the traditional Gibbs surface excess (Figure 2),
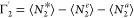
1bwhich is the presence of ⟨*N*_2_^*e*^⟩, signifying the number of sorbate in the solid sorbent side, in eq 1b. In the conventional approach, when eq 1b is applied for isotherms, ⟨*N*_2_^*e*^⟩ = 0 is commonly assumed. Consequently, when absorption of sorbate into sorbent takes place or cannot be ruled out, the conventional approach (eq 1b) cannot be applied.^1,4^ Under this assumption, Γ_2_^′^ (eq 1b) becomes formally identical to Γ_2_ (eq 1a). However, there are four reasons why Γ_2_ is advantageous over the Gibbsian Γ_2_^′^. First, Γ_2_, which does not exclude absorption, has a wider applicability than Γ_2_^′^. Second, Γ_2_ (eq 1a) offers a clearer mathematical analogy to the solution/solid relative surface excess, denoted by Γ_2_^(1)^ and defined as^2,15^
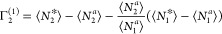
2because eq 2 involves two systems (* and *a*) just like eq 1a, while the traditional Gibbs formalism (eq 1b) contains three systems (*, *e*, and *a*). We emphasize here that the ensemble averaging in eq 2 for the system (*) incorporates the structural changes of sorbate and solvent molecules caused by the presence of the interface. Third, in the absence of the solvent (species 1), Γ_2_^(1)^ (eq 2) for liquid/solid becomes identical to Γ_2_ (eq 1a) for vapor/solid but not to the Gibbsian Γ_2_^′^ (eq 1b). Fourth, Γ_2_ and Γ_2_^(1)^ are in closer accordance with the experimental practice of isotherm determination.^2,4^ The standard experimental procedure for liquid sorption measures the reduced surface excess, Γ_2_^(*n*)^, from the change in solution composition upon the introduction of the sorbent;^2^ Γ_2_^(1)^ is determined via Γ_2_^(1)^ = Γ_2_^(*n*)^/*x*_2_^*a*^ (where *x*_1_^*a*^ is the mole fraction of solvent in the reference system *a*).^2^ The standard practice in gas sorption measures Γ_2_ (including absorption) rather than carrying out additional experiment to determine ⟨*N*_2_^*e*^⟩, required for Γ_2_^′^.^18,20,21^

Thus, adopting Γ_2_ and Γ_2_^(1)^ as the analogous measures of sorption is not only consistent with the standard experimental practice but also simpler, which requires the sole assumption of sorbent indissolubility (i.e., ⟨*N*_*e*_^*a*^⟩ = 0) for linking Γ_2_ and Γ_2_^(1)^ to the thermodynamics of vapor/solid and solution/solid interfaces (Supporting Information: Section A).^16^ We emphasize that defining surface excesses via eqs 1a and 2 are advantageous also in incorporating *absorption* into sorbents.^16^ In addition to its simplicity, this approach can handle arbitrary geometry and porosity without a need to define a coordinate system for the introduction of the Gibbs dividing surface (Supporting Information: Section A).

To summarize, the vapor/solid and solution/solid surface excesses have been defined analogously, in closer accordance with the standard experimental practice and with significant ease for dealing with interfacial porosity and sorbate absorption.

### Fluctuation Equations

Having introduced the surface excess for gas and liquid sorption analogously (Γ_2_ and Γ_2_^(1)^ in eqs 1a and 2), here we also establish sorbate number fluctuations analogously as the fundamental relationships for deriving isotherm equations. In doing so, we denote the number deviation (from the mean) via *δN_i_* = *N_i_* – ⟨*N*_*i*_⟩ for species *i*. What we present below is a generalization of fluctuation solution theory,^5,22−24^ initiated by Kirkwood and Buff,^25^ to interfaces.

#### Gas

**Figure 3:**
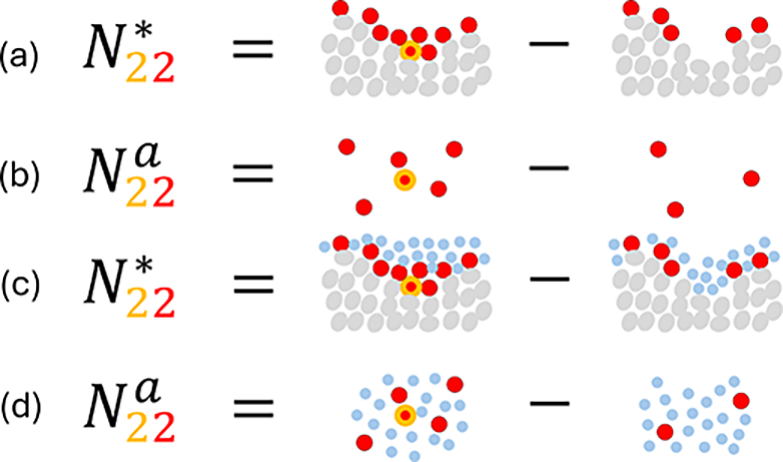
Excess numbers of sorbates (red spheres) around a probe sorbate (denoted by the orange circle): (a) at the vapor/solid interface (*N*_22_^*^, eq 3b), (b) in the sorbate vapor reference system (*N*_22_^*a*^, eq 3b), (c) at liquid/solid interface (*N*_22_^*^, eq 4b), and (d) in the solution reference system (*N*_22_^*a*^, eq 4b). The excess numbers are the key descriptors of the sorbate–sorbate interaction, which is related to the gradient of a sorption isotherm via eqs 3c and 4c. Note that the volume and solvent number define the sizes of the system and reference for gas and liquid sorptions, respectively.

Differentiating eq 1a with respect to ln *a*_2_ yields
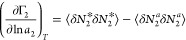
3awhere ⟨*δN*_2_^*^δN_2_^*^⟩ and ⟨*δN*_2_^*a*^*δN*_2_^*a*^⟩ represent the sorbate number fluctuation in the system and sorbate vapor reference, respectively.^21,26^Equation 3a can be rewritten using the sorbate excess numbers (Figure 3), defined as^21,26^
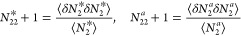
3binto
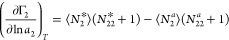
3cwhich is the excess number relationship for vapor/solid interfaces (Figure 3).^17,21^

#### Liquid

To formulate the excess number relationship for sorption from solution in a form mathematically analogous to the gas counterpart (eq 3c), we adopt the {*T*, *P*, *N*_1_, μ_2_} ensemble, by taking advantage of the equivalence of ensembles (via *N*_1_ = ⟨*N*_1_⟩ in eq 2 to introduce the constancy of *N*_1_) and the ease of transformation between ensembles via a statistical approach.^27,28^ Under this condition, differentiating eq 2 with respect to ln *a*_2_ yields
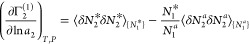
4awith the subscripts {*N*_1_^*^} and {*N*_1_^*a*^} introduced as the shorthand for {*T*, *P*, *N*_1_^*^, μ_2_} and {*T*, *P*, *N*_1_^*a*^, μ_2_} ensembles for the system and sorbate reference in which ensemble averaging has been carried out. Note that the number fluctuations in eq 4a reflect the potential of mean force between the sorbates that are mediated by the solvent (species 1).^8−11^ Introducing the excess numbers analogously to the vapor/solid systems (Figure 3), via^15^
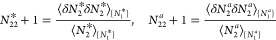
4bwe can express eq 4a in terms of the excess numbers (eq 4b), as (Figure 3)^15^
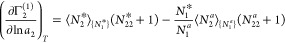
4cThus, the excess number relationship for vapor/solid (eq 3c) and solution/solid (eq 4c) systems are analogous, for which the adoption of the {*T*, *P*, *N*_1_, μ_2_} ensemble was crucial.^15,17^

## Gas and Liquid Isotherms Are Analogous

Our second objective is to derive the gas and liquid solution isotherms systematically from a universal theoretical foundation furnished in the previous section, thereby establishing an analogy between the two classes of isotherms (objective II). Our goal is to extend the powerful gas–solute analogy for the theory of liquids^5−11^ to sorption isotherms.

### Gas Isotherm

Based on the theoretical foundation summarized above, we derive here the ABC isotherm for gas sorption, which contains the Langmuir, BET (Brunauer–Emmett–Teller), and GAB (Guggenheim–Anderson–De Boer) isotherms yet without their overly idealized assumptions, directly from the gas/solid excess number relationship (eq 3c). To do so, we rewrite eq 3c as^21^
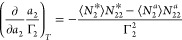
5aWe expand the right-hand side of eq 5a in terms of the sorbate activity, *a*_2_, as
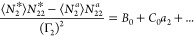
5bwhich is referred to as the characteristic equation,^17,19^ with the parameters *B*_0_ and *C*_0_.^15^ Integrating eq 5a, in combination with eq 5b, leads to the following ABC isotherm:^18−21^
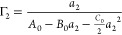
5cwhich was named after its parameters. The parameters of the ABC isotherm are expressed in terms of the numbers and number correlations, via^18−21^
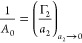
6a
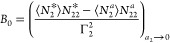
6band *C*_0_ with a more complex expression involving ternary correlations.^18,20^ We emphasize that the *a*_2_ → 0 limit in eqs 6a and 6b comes from the Maclaurin expansion (eq 5b) that the ABC isotherm is founded upon. The functional shape of eq 5c makes it clear that it is a generalization of the Langmuir, BET, and GAB isotherms.^18−21^ The general statistical thermodynamic nature of the parameters, defined solely in terms of the numbers and number correlations without any model assumptions, shows that the ABC isotherm is free from the overly idealized assumptions of previous isotherm models.

### Liquid Solution Isotherm

The ABC isotherm for the solution isotherm can be derived directly from the solution/solid excess number relationship (eq 4c), in a manner analogous to that for the gas/solid counterpart, by virtue of the constant *N*_1_ ensemble adopted in eq 4c. Just as in gas isotherms, first, we rewrite the excess number relationship (eq 4c) as^15^
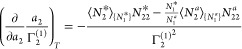
7aWe expand the right-hand side of eq 7a in terms of sorbate activity *a*_2_, as
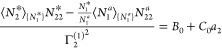
7bwhich is the characteristic equation for solution isotherms with the parameters *B*_0_ and *C*_0_.^15^ Integrating eq 7a with eq 7b yields the following ABC isotherm for the solution phase:^15^
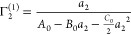
7cThe parameters of the ABC isotherm (eq 7c) are defined via the ensemble averages of numbers and number correlations, via
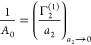
8a
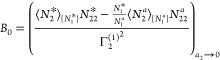
8bwith a more complex form, involving ternary correlations, for *C*_0_.^15^ The parameters of the ABC isotherm for liquid solutions are defined purely in terms of the general (model-free) statistical thermodynamic expressions involving ensemble averages without any need for any overly idealized assumptions that were invoked in the past when simply adapting the gas isotherm models for solutions.^15^

### Interfacial Locality as the Universal Language

The ABC isotherms for gases (eq 5c) and solutions (eq 7c) are founded on the common principle of interfacial locality; i.e., the effect of interface on sorbate structure is confined within a finite distance from the interface on the bulk sorbate side, tending to the bulk structure at larger distances. Formulating isotherms on interfacial locality has an additional advantage: establishing a theoretical analogy between sorption on interfaces and the solvation of molecules. In addition, Supporting Information: Section B makes it clear that the excess number relationships (eqs 3c and 4c) and the characteristic equations (eqs 5b and 7b) are local quantities; hence, the ABC isotherm parameters are also local for gases and solutions. Our common foundation contrasts with the previous isotherm models that involved separate foundations: binding sites for gases and surface phase for liquids.

## Gas and Liquid Isotherms Can Be Simplified Analogously

### “Surface Phase” Can Be Introduced for Both Gas and Liquid Isotherms

Having established the gas and solution isotherms on a theoretical foundation of interfacial locality analogous to solvation, here we simplify the gas and solution isotherms by introducing the “surface phase” in an analogous manner (Objective III). Our objective here is threefold: (III-i) to provide the analogous approximate treatments of gas and solution isotherms by introducing the “surface phase”, which was chiefly to solution isotherms, also to gas isotherms; (III-ii) to introduce the “actual amount sorbed” systematically for gas and solution isotherms clarifying the interpretation of the ABC isotherm parameters; (III-iii) to establish the novel interpretive tool, isotherm multiplicativity,^29^ for gas and solution isotherms when they are dominated by the actual amount sorbed.

#### Gas

Gas/solid isotherms have been measured via gravimetry, which directly measures the amount of sorption ⟨*n*_2_^*^⟩ (i.e., the quantity of sorbates associated locally with the sorbent, denoted throughout this Perspective by the lowercase *n*_2_^*^),^4^ namely,
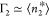
9awhich means neglecting the reference states while introducing the “interface” explicitly as the region of volume *v* within which the deviation from the bulk (reference state) is confined.^20^ This means that Γ_2_, defined via eq 1a, is an approximation for the gravimetrically measured ⟨*n*_2_^*^⟩, which is valid under sufficiently strong sorption (see the Supporting Information, section A of ref (21)). Under this condition, the ABC isotherm for gas sorption (eq 5c) can be simplified as
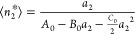
9bUsing eq 9a in conjunction with eqs 5c, 6a, and 6b leads to a simplified expression of the isotherm parameters. The parameter *A*_0_ can now be linked to the interface/bulk partition coefficient,  (where ⟨*n*_2_^*a*^⟩ is the number of sorbates in the bulk reference state with the same volume *v* as the interface),
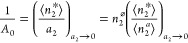
9cby taking advantage of *a*_2_ = ⟨*n*_2_^*a*^⟩/⟨*n*_2_^⌀^⟩ with ⟨*n*_2_^⌀^⟩ as the number of sorbates in the saturated reference system within *v*.^18,20,21^ The interpretation of the parameter *B*_0_ can also be simplified. To this end, it is convenient to deal with −1/*B*_0_ instead, because it is the saturation value of the isotherm (eq 9b) under *C*_0_ = 0, which is the generalization of the Langmuir isotherm.^19^ Under eq 9a, eq 6b simplifies to^18,20,21^
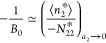
9dLet us clarify the physical meaning of eq 9d. The key is −*N*_22_^*^; since *N*_22_^*^ is the excess number of sorbates around a probe sorbate, −*N*_22_^*^ is the deficit number, i.e., the number of sorbates excluded by the presence of a probe sorbate. Therefore, according to eq 9d, the saturating capacity of the AB isotherm (eq 9b with *C*_0_ = 0) is the amount of sorption (⟨*n*_2_^*^⟩) per deficit number.^18,20,21^

Thus, we have introduced the surface phase for the analysis of gas isotherms, which enabled us to interpret the isotherm parameters in terms of the interface/gas partition coefficient, amount of sorption, and sorbate excess number in the surface phase.^18,20,21^ The clarity here contrasts with the previous attempts based on the hypothetical equations of states (EOS) for the interfacial “phase”,^1,30^ in which the simplest EOS led to the Volmer^31^ and Hill–de Boer^32,33^ models instead of rederiving the Langmuir, BET, and GAB models.

#### Solution

By introducing the “surface phase” with volume *v*, Γ_2_^(1)^, can be expressed as
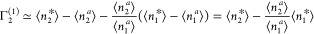
10awhere *n*_i_^*^ and *n*_*i*_^*a*^ express the numbers of species *i* within the surface phase and the bulk reference state with volume *v*, respectively.^15^ Note that the only approximation made in deriving eq 10a from eq 2 is the introduction of the interfacial volume. This enables us to introduce surface/bulk partitioning of sorbate and solvent, ⟨*n*_2_^*^⟩/⟨*n*_2_^*a*^⟩ and ⟨*n*_2_^*^⟩/⟨*n*_2_^*a*^⟩, through which eq 10a can be rewritten as
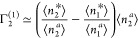
10bEquation 10b can be simplified further when Γ_2_^(1)^ can be approximated as the actual amount adsorbed at the interface,^15^ i.e.,
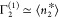
10cwhich has long been assumed, despite the admonishment of IUPAC, until the recent establishment of its theoretical basis as well as the quantitative condition for its validity.^16^ We emphasize that eq 10c, rather than eq 10a, corresponds to the gas sorption counterpart, eq 9a.

Under this condition (eq 10c), we will show that the solution isotherm (eq 7c) reduces to the form analogous to the simplified gas isotherm (eq 9b), facilitating the interpretation of the isotherm parameters. To do so, let us note that for a “dilute ideal” solution, *a*_2_ ≃ *x*_2_ = ⟨*n*_2_^*a*^⟩/⟨*n*_1_^*oa*^⟩ (with *n*_1_^*oa*^ being the concentration of pure solvent) applies, which simplifies the interpretation of *A*_0_,
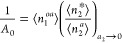
10das the surface/bulk partition coefficient of the sorbate, ⟨*n*_2_^*^⟩/⟨*n*_2_^*a*^⟩. Under this approximation, the saturation capacity, −1/*B*_0_, has a form mathematically analogous to the simplified vapor isotherm (eq 9d)
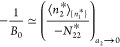
10eas the amount of sorption  per sorbate deficit number (−*N*_22_^*^), just like for the simplified vapor isotherm (eq 9d). Such a simple interpretation, in a form analogous to that of gas sorption, has been made possible by virtue of the constant *N*_1_ ensemble.

Thus, we have shown that an analogous formulation of gas and solution isotherms applies even when the “surface phase” is introduced to simplify them, thereby fulfilling objectives III-i and ii.

### Multiplicativity for Gas and Liquid Isotherms

Here, we show that gas and solution sorption obey the same fundamental equation when they are approximated by the actual amount sorbed (eqs 9a and 10c) and that isotherm multiplicativity applies, which can lead to novel insights into sorption mechanisms (objective III-iii). When Γ_2_ for gas sorption and Γ_2_^(1)^ for the solution can be approximated via the actual amount sorbed, ⟨*n*_2_^*^⟩ (see eqs 9a and 10c), eqs 3c and 4c (with ⟨*n*_2_^*^⟩ instead of ⟨*N*_2_^*^⟩) lead to a common fundamental equation for gas and solution isotherms^16,17^
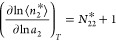
11aThis common foundation is the basis for the novel property of an isotherm: isotherm multiplicativity.^29^ This can be seen by rewriting eq 11a as
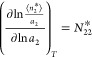
11bEquation 11b inspires the following isotherm multiplicativity rule: when the excess number is additive,
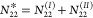
11cthe isotherm is multiplicative
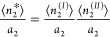
11dconsisting of the unconditional (*I*) and conditional (*II*) sorption processes.^29^

As the simplest example, let us consider a multiplication of the two AB isotherms (i.e., the ABC isotherms with *C* = 0), namely,
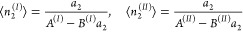
12aApplying isotherm multiplicativity (eq 11d) yields
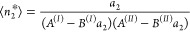
12bThe denominator is a product of two linear terms in *a*_2_, which is reminiscent of the BET and GAB isotherms and has the following functional form:
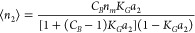
12cwhere *n*_*m*_ is the monolayer capacity, *C*_*B*_ is the BET constant, and *K*_*G*_ is the GAB constant, respectively; the BET model is a special case of GAB (eq 12c) with *K*_*G*_ = 1. A comparison between eqs 12b and 12c shows that the BET/GAB isotherm is a special case of the multiplicative isotherm (eq 12b) via
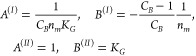
12d

**Figure 4:**
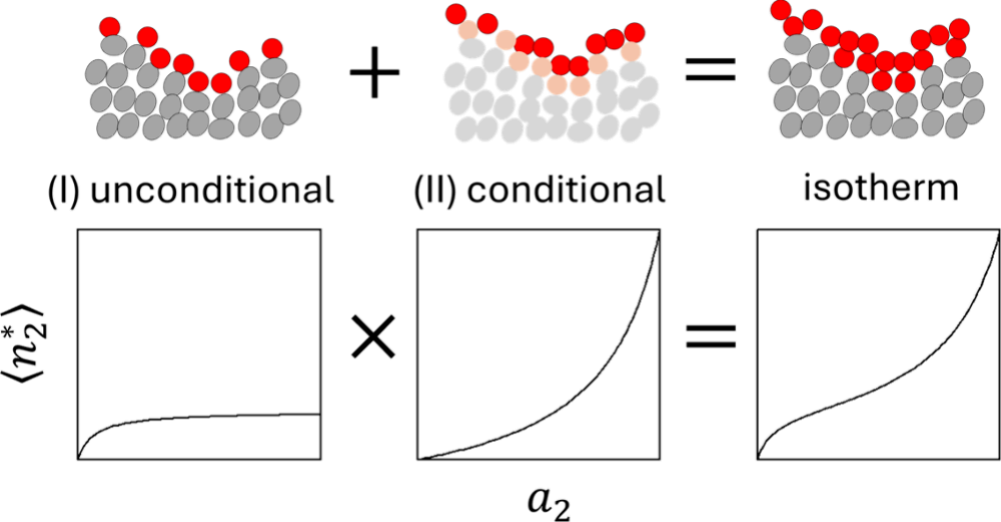
BET/GAB isotherm as the multiplicative isotherm consisting of (I) saturating adsorption (unconditional process) and (II) attractive sorbate–sorbate interaction (conditional process) in which the sorbate molecules sorbed at the interface in (I) attract further sorbate molecules. This mechanism is a generalization of the monolayer–multilayer mechanism for BET/GAB.

The above realization that BET/GAB is a special case of isotherm multiplicativity leads to their new interpretation (Figure 4). To this end, the unconditional step, combining eqs 12c and 12d, can be expressed as
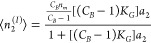
12ewhich has the mathematical form of the Langmuir isotherm with (*C*_*B*_ – 1)*K*_*G*_ corresponding to the “Langmuir constant” and *n*_*m*_*C*_*B*_/(*C*_*B*_ – 1) to the “monolayer capacity”. However, we emphasize that eq 12e as the AB isotherm (eq 12a) is not restricted to monolayer adsorption; it signifies the constancy of sorbate–surface attraction and sorbate–sorbate exclusion regardless of *a*_2_.^18^ The conditional step can be expressed as
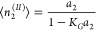
12fwith the form of a so-called “anti-Langmuir” isotherm, which, according to our recent paper,^18^ signifies sorbate–sorbate attraction at the interface. Thus, the BET/GAB model can be interpreted as the multiplicative process of (1) saturating adsorption (unconditional process) and (2) attractive sorbate–sorbate interaction (conditional process); the sorbate molecules sorbed at the interface attract further sorbate molecules, which is a generalization of the monolayer–multilayer mechanism for BET/GAB (Figure 4).

Our reinterpretation of BET/GAB may help rationalize why they are “less common in solution than in the gas phase”.^34^ Since the saturating AB isotherm (such as Langmuir or eq 12e) is observed frequently for solution isotherms, the less common occurrence of BET/GAB for solution is due to the relative rarity of the conditional process (eq 12f; rather than that of the multilayer formation^34^). In the solution, sorbate–sorbate interactions are mediated by the solvent molecules but not in the gas, which is the key difference between gas and solution isotherms. Hence, desolvation is not enhanced by localizing sorbates at the interface, which may be the reason that BET/GAB-like behavior is less common for solution isotherms.

## Moving Forward

### Universal Foundation of Sorption

The goal of this Perspective was to establish a common theory for gas and liquid isotherms analogously from their fundamentals, through isotherm equations, to their simplifications. This was achieved by rewriting the sorption theory via the statistical thermodynamic fluctuation theory, founded on the molecular distribution function as the universal measure for sorbate–sorbate interactions both for gases and for sorbate solutes mediated by the solvent.^5−11^ The signatures of sorption (i.e., surface excesses and excess numbers) for gases and liquids are not only analogous but also local, whose spatial contributions are restricted within a finite distance just like the solvation shell.^22,23,35^ This elevates sorption isotherms to the same level of universality as solution theory, where the analogy between gas and osmotic pressures serves as a powerful tool for interpretation.

### Practical Implications

The gas–liquid analogy in the theoretical foundation leads to isotherm equations usable not only for gas sorption but also for liquid solutions. When analyzing sorption isotherms, there is no longer any need to switch back and forth between different assumptions, such as (a) site-specific, layer-by-layer binding on a uniform surface for gas sorption and (b) “surface phase”^1−3^ and surface/bulk partition coefficients for sorption from solution. These overly idealized assumptions (e.g., (a) and (b))^14^ can be eliminated because our statistical thermodynamic isotherms are derived directly from the model-free concepts of surface excesses and number fluctuations.

### Decluttering Isotherm Models

Previously, isotherm models were derived on an individual basis, each based on a set of assumptions on the sorption mechanism and interfacial geometry. This has led to the proliferation of isotherm models, with more than 100 models listed in the literature for gas sorption alone.^36−42^ There is a need to reconsider whether it is productive to keep inventing new isotherm models. We propose to capture complex isotherms by combining simple isotherms, for which two approaches have so far been known: isotherm additivity and multiplicativity. Isotherm additivity can capture sorption isotherms on statistically independent surface patch types, such as heterogeneous pores^43^ and surfaces.^19^ Isotherm multiplicativity can reduce the BET and GAB models to the conditional process consisting of Types I and III isotherms, in addition to its track record in rationalizing the “anomalous” isotherm observed in membrane polymers.^29^

In summary, the theory of sorption has attained the same level of universality as enjoyed by the theory of liquid solutions^12,13^ where the gas–liquid analogy (i.e., virial expansion for gases and solutes) has long been established as the standard tool for analyzing experimental data.^5−11^ We hope that our approach to sorption isotherms will be applied to diverse isotherm classifications observed in sorption from solution and bring simplicity and clarity through the universal principles.

## Supporting Information Available

Analogous surface excesses for gases and solutions; locality of the excess numbers (PDF)
