# Supplementary material for: Gas and Liquid Isotherms: The Need for a Common Foundation
Source: Langmuir. 2025 Jan 21;41(4):2103–10. doi: 10.1021/acs.langmuir.4c04324 (PMC11803701; doi:10.1021/acs.langmuir.4c04324)
Supplement: Supplementary file 1 — la4c04324_si_001.pdf [file la4c04324_si_001.pdf]

Supporting information

Gas and Liquid Isotherms: The Need for a Common Foundation

Seishi Shimizu[1,*] and Nobuyuki Matubayasi[2]

[1]York Structural Biology Laboratory, Department of Chemistry, University of York, Heslington, York YO10 5DD, United Kingdom.

[2]Division of Chemical Engineering, Graduate School of Engineering Science, Osaka University, Toyonaka, Osaka 560-8531, Japan.

*Email: seishi.shimizu@york.ac.uk

Table of Contents

A: Analogous Surface Excesses for Gases and Solutions. p S1.

B. Locality of the Excess Numbers. p S4.

A: Analogous Surface Excesses for Gases and Solutions

Here, we present an alternative to the standard approach to the Gibbs adsorption isotherm. Instead of a trio of Gibbs-Duhem equations for the system (*) and the reference systems on the sorb*a*te (a) side (that are expressed via superscripts) and the sorbent side, we consider a pair of the Gibbs-Duhem equations for the system ($*$) and the reference system on the sorbate side (a) as[1,2]

$$\langle N_e^*\rangle d\mu_e + \langle N_1^*\rangle d\mu_1 + \langle N_2^*\rangle d\mu_2 - V^* dP = 0 \qquad \text{(A1a)}$$

$$\langle N_e^a\rangle d\mu_e + \langle N_1^a\rangle d\mu_1 + \langle N_2^a\rangle d\mu_2 - V^a dP = 0 \qquad \text{(A1b)}$$

where the temperature is kept fixed ($dT = 0$), $\langle N_e^*\rangle$, $\langle N_1^*\rangle$, and $\langle N_2^*\rangle$ are the numbers of sorbent, solvent, and sorbate in the system, $\langle N_e^a\rangle$, $\langle N_1^a\rangle$, and $\langle N_2^a\rangle$ are the numbers of the corresponding species in the reference system, V^* and V^a are the volumes of the system and reference solution phase, P is the pressure, and μ_i is the chemical potential of species i, respectively.[2] Taking the reference system a as the bulk solution, the pair (eqs A1a and A1b), in the present form, describes a solution/solid interface. By setting $\langle N_1^*\rangle = \langle N_1^a\rangle = 0$, eqs A1a and A1b turns into a gas/solid interface. Consequently, eqs A1a and A1b are applicable to solution/solid and gas/solid interfaces.

The only assumption introduced in this formalism is that the sorbent molecules do not dissolve into the solution phase (i.e., phase a), i.e.,[1]

$$\langle N_e^a \rangle = 0 \tag{A1c}$$

Under this condition (eq A1c), subtracting eq A1b from eq A1a leads to

$$\langle N_e^* \rangle d\mu_e + (\langle N_1^* \rangle - \langle N_1^a \rangle) d\mu_1 + (\langle N_2^* \rangle - \langle N_2^a \rangle) d\mu_2 - (V^* - V^a) dP = 0 \tag{A2}$$

Equation A2 is the common foundation for gas and liquid isotherms. For liquid isotherms, P and μ_2 are independent thermodynamic variables. Consequently, taking the μ_2-derivative of eq A2 under constant P and T yields

$$-\left(\frac{\partial \mu_e}{\partial \mu_2}\right)_{T,P} = \frac{\langle N_2^* \rangle - \langle N_2^a \rangle}{\langle N_e^* \rangle} + \left(\frac{\partial \mu_1}{\partial \mu_2}\right)_{T,P} \frac{\langle N_1^* \rangle - \langle N_1^a \rangle}{\langle N_e^* \rangle} \tag{A3a}$$

Here, $\left(\frac{\partial \mu_1}{\partial \mu_2}\right)_{T,P}$, a bulk quantity, can be evaluated via eqs A1b and A1c, as

$$\left(\frac{\partial \mu_1}{\partial \mu_2}\right)_{T,P;\langle N_e^a \rangle = 0} = -\frac{\langle N_2^a \rangle}{\langle N_1^a \rangle} \tag{A3b}$$

Combining eqs A3a and A3b leads to

$$-\left(\frac{\partial \mu_e}{\partial \mu_2}\right)_{T,P} = \frac{\langle N_2^* \rangle - \langle N_2^a \rangle}{\langle N_e^* \rangle} - \frac{\langle N_2^a \rangle}{\langle N_1^a \rangle} \frac{\langle N_1^* \rangle - \langle N_1^a \rangle}{\langle N_e^* \rangle} \equiv \Gamma_2^{(1)} \tag{A4}$$

Equation A4 is the relative surface excess, defined per unit mass of sorbent ($\langle N_e^* \rangle$), which is in line with the experimental convention.[3] (The condition for convergence of the individual surface excesses of solvent and sorbate will be discussed at the end of this section.)

For gas sorption, P and μ_2 are no longer independent since the number of components is smaller by one for the gas/solid system than for the liquid/gas. In the following, we will derive the gas sorption counterpart of eq A4 via a discussion parallel to the previous paragraph. Taking a μ_2-derivative of eq A2, under $\langle N_1^* \rangle = \langle N_1^a \rangle = 0$ for the gas/solid interface, leads to

$$-\left(\frac{\partial \mu_e}{\partial \mu_2}\right)_T = \frac{\langle N_2^* \rangle - \langle N_2^a \rangle}{\langle N_e^* \rangle} - \frac{V^* - V^a}{\langle N_e^* \rangle}\left(\frac{dP}{\partial \mu_2}\right)_T \tag{A5a}$$

which corresponds to eq A3a, with the difference of P no longer being an independent variable in the Gibbs phase rule. Next, we eliminate $\left(\frac{dP}{\partial \mu_2}\right)_T$, which can be achieved by using eqs A1b and A1c, in conjunction with under $\langle N_1^* \rangle = \langle N_1^a \rangle = 0$, which leads to

$$\left(\frac{dP}{\partial \mu_2}\right)_{T;\langle N_e^a \rangle = 0} = \frac{\langle N_2^a \rangle}{V^a} \tag{A5b}$$

as the gas/solid counterpart of eq A3b. Combining eqs A5a and A5b yields

$$-\left(\frac{\partial \mu_e}{\partial \mu_2}\right)_T = \frac{\langle N_2^* \rangle}{\langle N_e^* \rangle} - \frac{V^*}{V^a}\frac{\langle N_2^a \rangle}{\langle N_e^* \rangle} \tag{A6}$$

which is the gas/solid version of eq A4. The right-hand side of eq A6, via the introduction of the bulk sorbate concentration,

$$c_2^a = \langle N_2^a \rangle / V^a \quad \text{(A7a)}$$

can be rewritten as

$$-\left(\frac{\partial \mu_e}{\partial \mu_2}\right)_T = \frac{\langle N_2^* \rangle - c_2^a V^*}{\langle N_e^* \rangle} \quad \text{(A7b)}$$

In eq A7b, $\langle N_2^* \rangle - c_2^a V^*$ signifies the sorbate excess number, in the presence of the sorbate, relative to the bulk sorbate system of the same volume, $c_2^a V^*$. (The necessary condition for its convergence from a molecular distribution perspective will be discussed at the end of this section.) The contributions to $\langle N_2^* \rangle - c_2^a V^*$ can be dissected by rewriting eq A6 as

$$-\left(\frac{\partial \mu_e}{\partial \mu_2}\right)_{T,P} = \Gamma_2 - \frac{(V^* - V^a) c_2^a}{\langle N_e^* \rangle} \quad \text{(A8a)}$$

where the first term is the surface excess defined as

$$\Gamma_2 = \frac{\langle N_2^* \rangle - \langle N_2^a \rangle}{\langle N_e^* \rangle} \quad \text{(A8b)}$$

which is analogous to the first term of $\Gamma_2^{(1)}$ of the solution/solid theory (eq A4) and the second term, $(V^* - V^a) c_2^a$, signifies buoyancy of the sorbent caused by the exclusion of bulk sorbate gas, which is taken into account ("buoyancy correction"[4,5]) in the gravimetric determination of gas/solid isotherms.

In the main text, surface excesses $\langle N_1^* \rangle - \langle N_1^a \rangle$ and $\langle N_2^* \rangle - \langle N_2^a \rangle$ are defined per unit mass of sorbent, hence the division by $\langle N_e^* \rangle$ is omitted throughout.

Before concluding this section, we must clarify the convergence condition for surface excesses from the underlying molecular distributions. First, for gas sorption, in order for $\langle N_2^* \rangle - c_2^a V^*$ in eq A7b to converge, it must be expressed as

$$\langle N_2^* \rangle - c_2^a V^* = \int_{\vec{r} \in V^*} d\vec{r} [\rho_2^*(\vec{r}) - c_2^a] \quad \text{(A9)}$$

where $\rho_2^*(\vec{r})$ is the local sorbate density at the position $\vec{r}$, with the range of integration over V^*. For eq A9 to converge, $\rho_2^*(\vec{r}) \to c_2^a$ at the locations sufficiently far from the interface. Second, to guarantee the convergence of individual surface excesses of solvent and sorbate, we rewrite the relative surface excess (eq 2) as

$$\Gamma_2^{(1)} = \langle N_2^* \rangle - c_2^a V^* - \frac{\langle N_2^a \rangle}{\langle N_1^a \rangle} (\langle N_1^* \rangle - c_1^a V^*) \quad \text{(A10)}$$

Then we can introduce the local $\rho_i^*(\vec{r})$ and bulk c_i^a densities for both solvent ($i = 1$) and sorbate ($i = 2$), through which we can introduce the individual surface excess as

$$\langle N_i^* \rangle - c_i^a V^* = \int_{\vec{r} \in V^*} d\vec{r} [\rho_i^*(\vec{r}) - c_i^a] \quad \text{(A11)}$$

as the generalization of the gas sorption formalism (eq A9). For the individual surface excess defined via eq A11 to converge, $\rho_2^*(\vec{r}) \to c_2^a$ at the locations sufficiently far from the interface.

B. Locality of the Excess Numbers.

Here, we show that the excess number relationships (eqs 3c and 4c) are "local", signifying (by extending the definition given for solvation[6–9]) that they come from the molecules located within a finite distance from the interface. The analogy between excess number relationships for vapor/solid and solution/solid systems (eqs 3c and 4c) will facilitate the parallel demonstration of localness. In contrast to the mathematical complication caused by the conventional definition of the surface excess (eq 1b) adopted in our previous paper,[10] the only postulate required here is that the sorbate gas or sorbate-solvent mixture tend to their bulk behaviors sufficiently away from the interface, namely, (i) the sorbate-interface and solvent-interface distribution functions tend to their bulk values and (ii) sorbate-sorbate distribution function tends to its functional shape in the bulk.

Gas. First, $\Gamma_2 = \langle N_2^* \rangle - \langle N_2^a \rangle$ (eq 1a) is a local quantity because sorbate distribution deviates from the bulk value only within a finite distance from the interface on the gas side, i.e., postulate (i). Second, $\langle \delta N_2^* \delta N_2^* \rangle - \langle \delta N_2^a \delta N_2^a \rangle$ (eq 3a) is a local quantity because sorbate-sorbate distribution deviates from the bulk only within a finite distance from the interface on the liquid side, i.e., postulate (ii). Third, $\langle N_2^* \rangle N_{22}^* - \langle N_2^a \rangle N_{22}^a$, which is the difference between the two local quantities, as can be seen via

$$\langle N_2^* \rangle N_{22}^* - \langle N_2^a \rangle N_{22}^a = (\langle \delta N_2^* \delta N_2^* \rangle - \langle \delta N_2^a \delta N_2^a \rangle) - \Gamma_2 \quad \text{(B1)}$$

This will play a crucial role in deriving the isotherm equation in the section entitled "Gas and Liquid Isotherms Can Be Simplified Analogously". Note that the locality of absorption contribution on the solid side comes from the finite penetration of sorbates into sorbent.

Liquid. First, $\langle N_1^* \rangle - \langle N_1^a \rangle$ and $\langle N_2^* \rangle - \langle N_2^a \rangle$ are local quantities because their local densities deviate from the bulk values only within a finite distance from the interface (postulate (i)). $\Gamma_2^{(1)}$, which is the linear combination of the two local quantities, is also local. Second, to show that $\left(\frac{\partial \Gamma_2^{(1)}}{\partial \ln a_2}\right)_T$ is a local quantity, it is convenient to rewrite eq 4c as

$$\left(\frac{\partial \Gamma_2^{(1)}}{\partial \ln a_2}\right)_T = \left(\langle \delta N_2^* \delta N_2^* \rangle_{\{N_1^*\}} - \langle \delta N_2^a \delta N_2^a \rangle_{\{N_1^a\}}\right) - \frac{N_1^* - N_1^a}{N_1^a} \langle \delta N_2^a \delta N_2^a \rangle_{\{N_1^a\}} \quad \text{(B2)}$$

On the right-hand side of eq B2, $\langle \delta N_2^* \delta N_2^* \rangle_{\{N_1^*\}} - \langle \delta N_2^a \delta N_2^a \rangle_{\{N_1^a\}}$ is a local quantity because the effect of the interface on sorbate-sorbate distribution is limited within a finite distance (postulate (ii)). $N_1^* - N_1^a$ is a local quantity because the effect of interface on solvent distribution is restricted within a certain distance (postulate (i)). Equation B2, being a linear combination of the two, is also local. Third, the following quantity, being a linear combination of the two local quantities, is local:

$$\langle N_2^* \rangle_{\{N_1^*\}} N_{22}^* - \frac{N_1^*}{N_1^a} \langle N_2^a \rangle_{\{N_1^{II}\}} N_{22}^a = \left(\langle \delta N_2^* \delta N_2^* \rangle_{\{N_1^*\}} - \frac{N_1^*}{N_1^a} \langle \delta N_2^a \delta N_2^a \rangle_{\{N_1^a\}} \right) - \Gamma_2^{(1)} \quad \text{(B3)}$$

The local nature of eq B3 will play an important role in deriving the isotherm equation for sorption from solution in the section entitled “Gas and Liquid Isotherms Can Be Simplified Analogously”.

References

(1) Shimizu, S.; Matubayasi, N. Cooperativity in Sorption Isotherms. *Langmuir* **2023**, *37* (34), 10279–10290. https://doi.org/10.1021/acs.langmuir.3c01243.

(2) Shimizu, S.; Matubayasi, N. Actual Amount Adsorbed as Estimated from the Surface Excess Isotherm. *Langmuir* **2024**, *40*, 1666–1673. https://doi.org/10.1021/acs.langmuir.3c02597.

(3) Everett, D. H. Reporting Data on Adsorption from Solution at the Solid/Solution Interface (Recommendations 1986). *Pure Appl. Chem.* **1986**, *58* (7), 967–984. https://doi.org/10.1351/pac198658070967.

(4) Thommes, M.; Kaneko, K.; Neimark, A. V; Olivier, J. P.; Rodriguez-Reinoso, F.; Rouquerol, J.; Sing, K. S. W. Physisorption of Gases, with Special Reference to the Evaluation of Surface Area and Pore Size Distribution (IUPAC Technical Report). *Pure Appl. Chem.* **2015**, *87*, 1051–1069. https://doi.org/10.1515/pac-2014-1117.

(5) Rouquerol, F.; Rouquerol, J.; Sing, K. S. W. Adsorption by Powders and Porous Solids, Second Edition; Elsevier: Amsterdam, 2013; pp 237–438.

(6) Matubayasi, N.; Reed, L. H.; Levy, R. M. Thermodynamics of the Hydration Shell. 1. Excess Energy of a Hydrophobic Solute. *J. Phys. Chem.* **1994**, *98* (41), 10640–10649. https://doi.org/10.1021/j100092a040.

(7) Matubayasi, N.; Levy, R. M. Thermodynamics of the Hydration Shell. 2. Excess Volume and Compressibility of a Hydrophobic Solute. *J. Phys. Chem.* **1996**, *100* (7), 2681–2688. https://doi.org/10.1021/jp951618b.

(8) Matubayasi, N.; Gallicchio, E.; Levy, R. M. On the Local and Nonlocal Components of Solvation Thermodynamics and Their Relation to Solvation Shell Models. *J. Chem. Phys.* **1998**, *109* (12), 4864–4872. https://doi.org/10.1063/1.477097.

(9) Levy, R. M.; Cui, D.; Zhang, B. W.; Matubayasi, N. Relationship between Solvation Thermodynamics from IST and DFT Perspectives. *J. Phys. Chem. B* **2017**, *121* (15), 3825–3841. https://doi.org/10.1021/acs.jpcb.6b12889.

(10) Shimizu, S.; Matubayasi, N. Fluctuation Adsorption Theory: Quantifying Adsorbate-Adsorbate Interaction and Interfacial Phase Transition from an Isotherm. *Phys. Chem. Chem. Phys.* **2020**, *22*, 28304–28316. https://doi.org/10.1039/D0CP05122E.
